# Hypertrophy of paravertebral muscles after epidural electrical stimulation shifted: A case report

**DOI:** 10.3389/fsurg.2022.936259

**Published:** 2022-07-27

**Authors:** Sipeng Li, Hongtao Rong, Zhenghao Hao, Rui Tan, Haijun Li, Tao Zhu

**Affiliations:** Department of Neurosurgery, Tianjin Medical University General Hospital, Tianjin, China

**Keywords:** epidural electrical stimulation, paravertebral muscles, 18F-fluorodeoxyglucose, spinal cord injury, case report

## Abstract

Epidural electrical stimulation (EES) has been used to improve motor function in patients with chronic spinal cord injury (SCI). The effect of EES on paravertebral muscles in patients with SCI has been unnoticed. We reported a case of paravertebral muscles hypertrophy after the electrode shifted in a patient with spinal cord injury. We also discussed possible mechanistic accounts for this occurs.

## Introduction

Spinal cord injury (SCI) can cause certain physiological and pathological changes in muscle fibers, among which muscle atrophy is the most critical (1, 2). Epidural electrical stimulation (EES) can activate the lumbar central pattern generator and allow patients with SCI to produce rhythmic movement, thus helping them achieve independent standing and physical stability (3–8). The paravertebral muscles are important for maintaining standing posture and balance. At present, no study has reported the effect of EES on the paravertebral muscles in patients with SCI. We reported a case of paravertebral muscles hypertrophy after the electrode shifted in a patient with spinal cord injury.

## Case report

**Figure 1:**
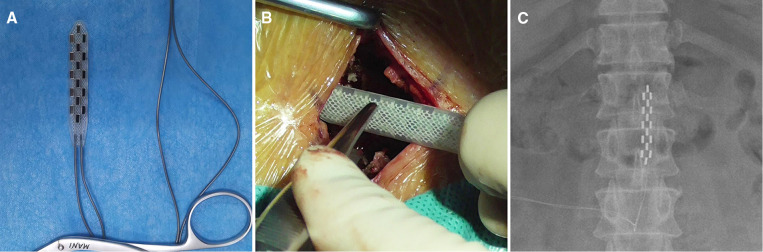
(**A**) Surgical electrode with 16 contacts; (**B**) Surgical electrode was implanted in the spinal canal; (**C**) The position of the electrode 6 months after the initial surgery.

**Figure 2:**
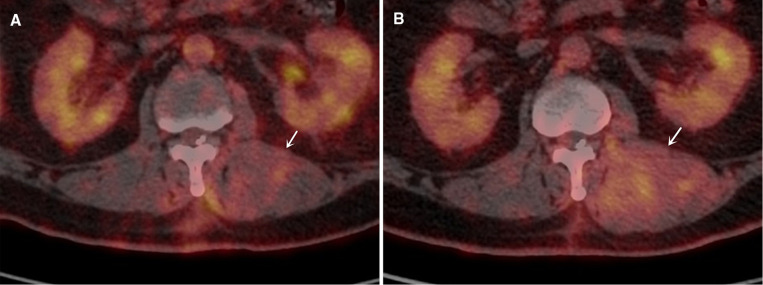
The images of paravertebral muscles on PET-CT at 3 and 6 months after surgery. (**A**) The image of paravertebral muscles on PET-CT at 3 months (white arrows indicates hypertrophic paravertebral muscles); (**B**) The image of paravertebral muscles on PET-CT at 6 months.

A 45-year-old man with T3-T4 level SCI due to a traffic accident 2 years ago. A surgical electrode with 16 contacts was implanted in the patient (Figures 1A,B). The parameter of stimulation was 4.4 V, 210 µm, and 60 Hz. However, the electrode shifted to the left after the surgery (Figure 1C), and the size of paravertebral muscles on this side significantly increased (Figure 2). Finally, we performed revision surgery 6 months after the initial surgery and readjusted the electrode position, and obtained bilateral paravertebral muscles tissue for biopsy.

We reviewed positron emission tomography-computed tomography (PET-CT) on this patient at 3 and 6 months postoperatively and obtained the cross-sectional areas (CSAs) of the bilateral paravertebral muscles. The fused PET-CT images showed that the CSAs of the bilateral paravertebral muscles in this patient at 6 months after surgery was significantly greater than that in corresponding slices at 3 months (Table 1), especially on the left side (Figure 2).

**Table 1:** Cross-sectional areas of paravertebral muscles.

Position	3 m(mm^2^)	6 m(mm^2^)
Right		
Paravertebral muscle above electrode	1235.981 ± 140.381	1356.38 ± 146.682
Paravertebral muscle below electrode	2077.079 ± 325.725	2223.721 ± 345.16
Left		
Paravertebral muscle above electrode	1153.491 ± 225.862	1255.616 ± 349.017
Paravertebral muscle below electrode	2410.068 ± 387.689	3188.735 ± 640.336

In addition, we further measured the mean standard uptake value (SUVmean) of 18F-fluorodeoxyglucose (18F-FDG) on the corresponding muscles. The SUVmean on the left paravertebral muscles below the electrode was significantly higher than that on the right at 6 months after surgery (Right: 0.229 ± 0.028, Left: 0.558 ± 0.181).

**Figure 3:**
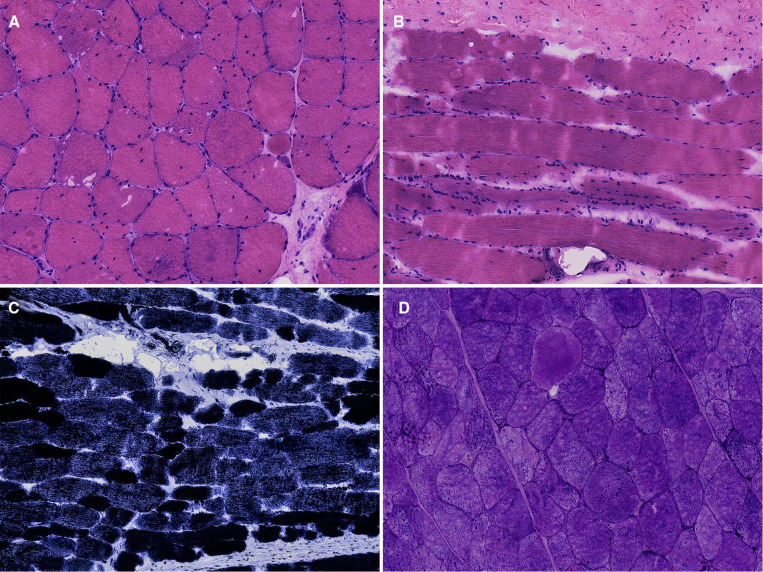
Biopsy of paravertebral muscles. (**A**) Hematoxylin-Eosin (HE) staining of the left paravertebral muscle, 20× ; (**B**) HE staining of the right paravertebral muscle, 20×; (**C**) Nicotinamide adenine dinucleotide tetrazolium oxidoreductase (NADH-TR) staining, 10×; (**D**) Periodic acid-schiff (PAS) staining, 20×.

The muscle biopsy results showed that the muscle fibers on both sides showed compensatory hypertrophy. Type II fibers were the main type of muscle fiber in the bilateral paravertebral muscles. The number of nuclei in some muscle fibers increased, moved inward to the sarcoplasmic cells, and were distributed in bundles, especially on the left side. PAS staining showed increased glycogen in the muscle fibers of the bilateral paravertebral muscles (Figure 3).

## Discussion

In SCI, joint activities of muscles are impaired due to denervated atrophy (9). Skeletal muscles are the foundation for maintaining posture and balance. EES promotes gait-related muscle recruitment by activating the lumbar central pattern generator, enabling patients with SCI to stand independently and achieve physical stability (3, 7, 10–12). EES can not only be used to treat traumatic SCI, but also has attractive prospects in the management of other forms of chronic SCI such as myelopathic changes induced by cervical spondylotic myelopathy or central cord syndrome.

This patient with SCI underwent EES and showed abnormal hypertrophy of the paravertebral muscle on one side due to electrode shift after surgery. The CSAs of the paravertebral muscles on both sides significantly increased after EES. Due to the asymmetric hypertrophy of the bilateral paravertebral muscles, we speculate that the influence of electrode movement on the bilateral muscles may be different. The SUVmean values of 18F-FDG on the left paravertebral muscles below the electrode were significantly higher than those on the right. Enhancing metabolism by EES might be a factor in promoting muscle hypertrophy.

Hypertrophy of the skeletal muscle occurs by promoting satellite cell activation and maintaining protein balance (13–16). Khodabukus et al. (14) found that the number of nuclei in the cross-section of the muscle bundle after electrical stimulation increased by 1.5 times compared with that in the non-stimulated group. In addition, the CSA of the muscle bundles in the electrically stimulated group increased by 1.8 times compared with that in the non-stimulated group. Electrical stimulation promotes human muscle hypertrophy, myotube maturation, and increased metabolism.

Some studies have shown that the mammalian target of rapamycin-1 can be activated by 4E-BP1 and S6K, thereby increasing protein synthesis (17–21). Phosphorylation of these proteins significantly increased with electrical stimulation at 10 and 1 Hz, which suggested that frequency-dependent hypertrophy of muscle fibers is the result of increased anabolic signals (14).

In addition, skeletal muscles have high plasticity, which is mainly reflected in the mutual transformation between different types of muscle fibers. These muscles can transform from fast twitch to slow twitch, which can be influenced by internal (genetics and nutrition) and external factors (22). At the waist, type I fibers account for 57% of superficial muscles and 63% of deep muscles (23). Hypertrophy of muscle fibers caused by electrical stimulation may be more obvious in type I muscle fibers (24). Muscle biopsy in this patient showed that the fiber types on both sides were almost dominated by type II muscle fibers. The electric pulse emitted by EES may induce transformation from type I to type II fibers.

In addition, the surgical electrode shift resulted not only in hypertrophy of paravertebral muscles, but also in an imbalance in the electrical impulses received by both sides. In order to prevent electrode shifted, the surgical electrode should be secured with a fixed anchor. The fixed anchor should be as close to the surgical electrode as possible. In addition, the position of surgical electrode after fixation was determined by intraoperative localization. If the electrode has shifted, revision surgery should be performed timely.

## Conclusion

In this case, the paravertebral muscles on the side of the electrode shifted were abnormally hypertrophy. We surmise that EES may affect the metabolism of these muscles and lead to skeletal muscle hyperplasia. In addition, EES may be involved in regulating transformation between different muscle fiber types. However, since only one case was reported, this conjecture requires further experimental confirmation.

## Data Availability

The raw data supporting the conclusions of this article will be made available by the authors, without undue reservation.
